# GOTrapper: a tool to navigate through branches of gene ontology hierarchy

**DOI:** 10.1186/s12859-018-2581-8

**Published:** 2019-01-11

**Authors:** Hezha Hassan, Siba Shanak

**Affiliations:** 1 Public Health Laboratory, Sulaimaniyah, Kurdistan Region 46001 Iraq; 2 Genome Informatics, Faculty of Technology and Center for Biotechnology (CeBiTec), Bielefeld University, Bielefeld, Germany; 3 Faculty of Sciences, Arab American University-Palestine, P.O Box 240, Jenin, Palestine

**Keywords:** Gene ontology, GO term refinement, Gene association

## Abstract

**Background:**

Gene Ontology (GO) is a useful resource of controlled vocabulary that provides information about annotated genes. Based on such resource, finding the biological function is useful for biologists to come up with different hypotheses and help further investigations of an experiment. The biological function for desired genes and gene associations is picked up from a randomly chosen list or through the analysis of differential gene expression. Many tools have been developed to utilize GO knowledge and cluster genes according to relevant biological functions. The retrieved GO terms include both specific and non-specific terms, which is not user-friendly in terms of data analysis. Thus one approach is still missing, which allows navigating through different levels of GO hierarchy manually.

**Result:**

We developed a tool, GOTrapper, which allows moving up or down to the very bottom of the GO hierarchy. This is performed manually by the user, based on an assigned threshold. This tool grabs the shared terms by the desired set of input genes of *Homo sapiens*. Here, two inputs are possible. “Within” is to find associated terms within one gene list, and “Between” is to find associated terms between two lists. The tool also provides the option to return the terms with the pre-selected evidence codes.

**Conclusion:**

GOTrapper is a user-friendly Java tool that helps the user move up and down the ontology tree, which leads to new hypotheses and devising new association of the input genes. It also allows returning terms of associated genes based on selected evidence codes. This tool can be accessed and is freely available at https://github.com/BioGeneTools/GOTrapper.

**Electronic supplementary material:**

The online version of this article (10.1186/s12859-018-2581-8) contains supplementary material, which is available to authorized users.

## Background

The Gene Ontology (GO) is a controlled vocabulary of gene annotations, which was founded in 1998 to provide interpretation of biological functions that are associated with individual genes [1, 2]. The GO terms were placed in a hierarchy and are structured as an acyclic directed graph. They are classified into three vocabularies: Biological Processes, Molecular Functions, and Cellular Components. Each term may have more than one parent and more than one child. Going down the graph, the terms get more specific.

Gene Ontology is a powerful tool and the largest resource for cataloguing gene function continuously used in data analysis and functional prediction. The usage of this tool by inexperienced users might draw false conclusions [3, 4]. In microarray and RNA-seq experiments, GO is used broadly as a tool to group genes as well as to determine term enrichment of different biological processes, molecular functions, and cellular components. This helps explain the biology of the sample conditions.

Many methods and tools have been developed to find terms and perform enrichment analysis from expression data. Nonetheless, there is still some hidden information needed to be revealed from GO, many redundant terms, and a lack of simplicity of the tools; especially for biologists.

One strategy to speculate the gene ontology list for an experiment is to find the enriched GO terms. Several statistical methods can be used for this analysis, such as the hypergeometric distribution, Fisher Exact test, and binomial test [5]. These methods serve in mining the statistically significant enriched terms and suffer from redundancies, due to the inclusion of less specific terms. There exist tools and algorithms that manipulate different techniques to reduce those redundancies, through removing parent terms from the list of enriched terms [6–8]. Still, the remaining ‘last’ children terms, which are extracted by the different statistical methods mentioned above, have important information that could be lost at the expanded level of the maintained children terms.

With increasing biological information and expanding ontological annotations, it is highly beneficial for biologists to have on hand tools to find the associations between the different desired sets of genes with less redundancy. Some tools have made this option available [6–14]. Some of these tools require the user to provide extra information such as *p*-values or expression data, which may be obtained from differential expression analysis. Other tools allow provisioning of the gene lists alone but they handle enrichment analysis. This causes the loss of specific associations between genes at the end of the branch of the GO tree.

There are also a number of tools, e.g.; web-based and plugins that provide a variety of functions but require internet connection or third-party software. This could be complicated or less helpful; especially for inexperienced users [8, 12–22].

It is important, especially for wet lab experimentalists, to utilize gene ontology resources in finding different gene associations and in making new hypotheses via the manual crawling through stages of hierarchy for the ontology. To our knowledge, there is no tool to provide such options.

In this paper, we developed a user-friendly, open source, and cross-platform tool to help experienced and inexperienced users in finding gene set associations. This tool offers manual navigation through ontology hierarchy by using the gene names only, and without the need for expression data, *p*-value, fold change calculations, or other inputs.

## Implementation

**Fig. 1:**
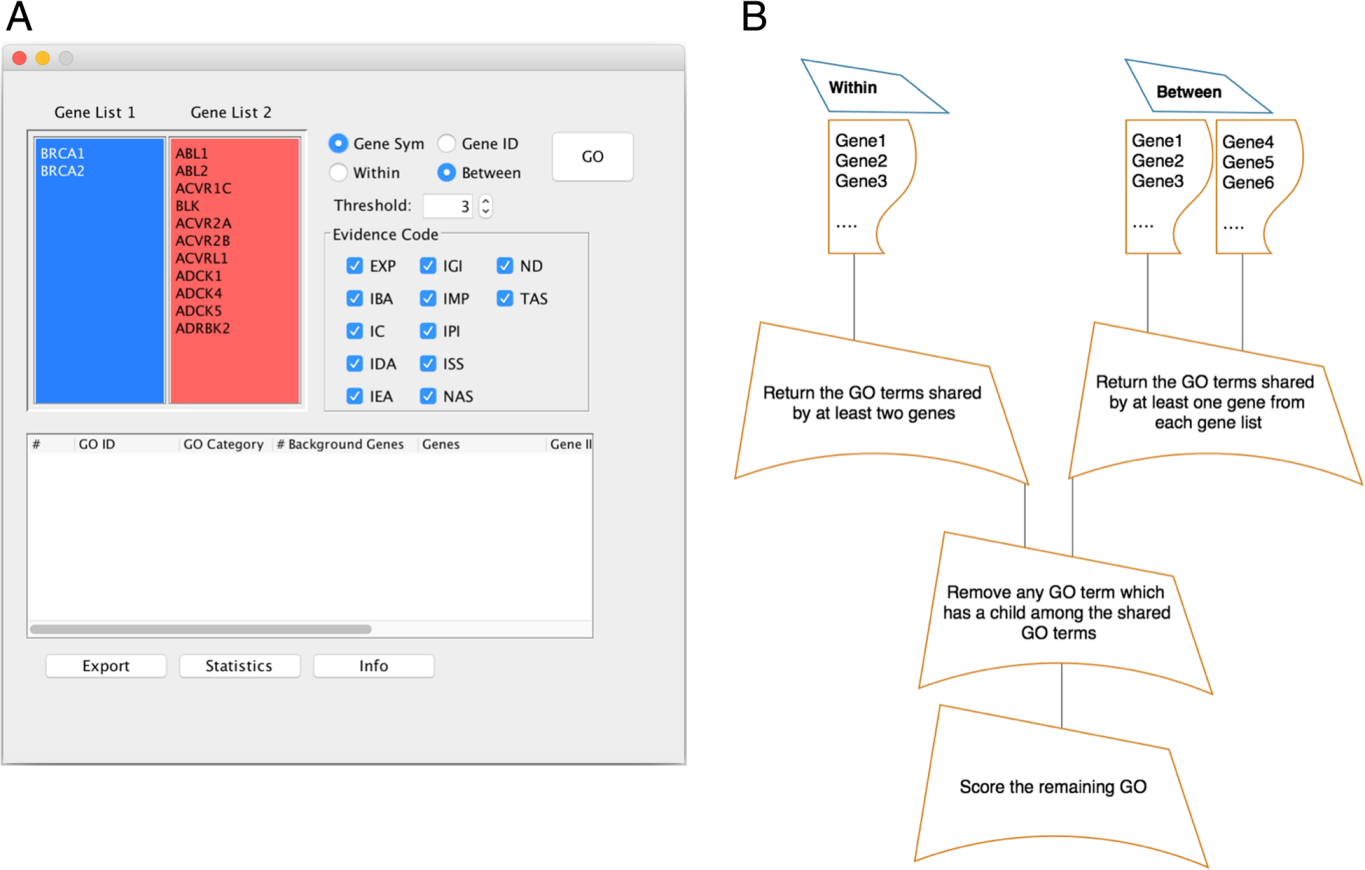
**a** The front-end of GOTrapper. **b** The workflow of GOTrapper tool. The main interface of the tool (**a**). Usage of the tool starts by choosing “Within” or “Between” options for a list or two lists of genes, respectively. After that, the shared GO terms are returned, which is followed by removing parent terms and scoring the refined terms. This workflow is shown in part (**b**)

Figure 1b depicts the workflow of the tool. The tool is open source and built in Java. GOTrapper does not rely directly on the GO database. It derives all the mapping and annotations from two databases, GO.db [23] and org.Hs.eg.db [24], from Bioconductor [25].

### Finding most specific GO terms

**Fig. 2:**
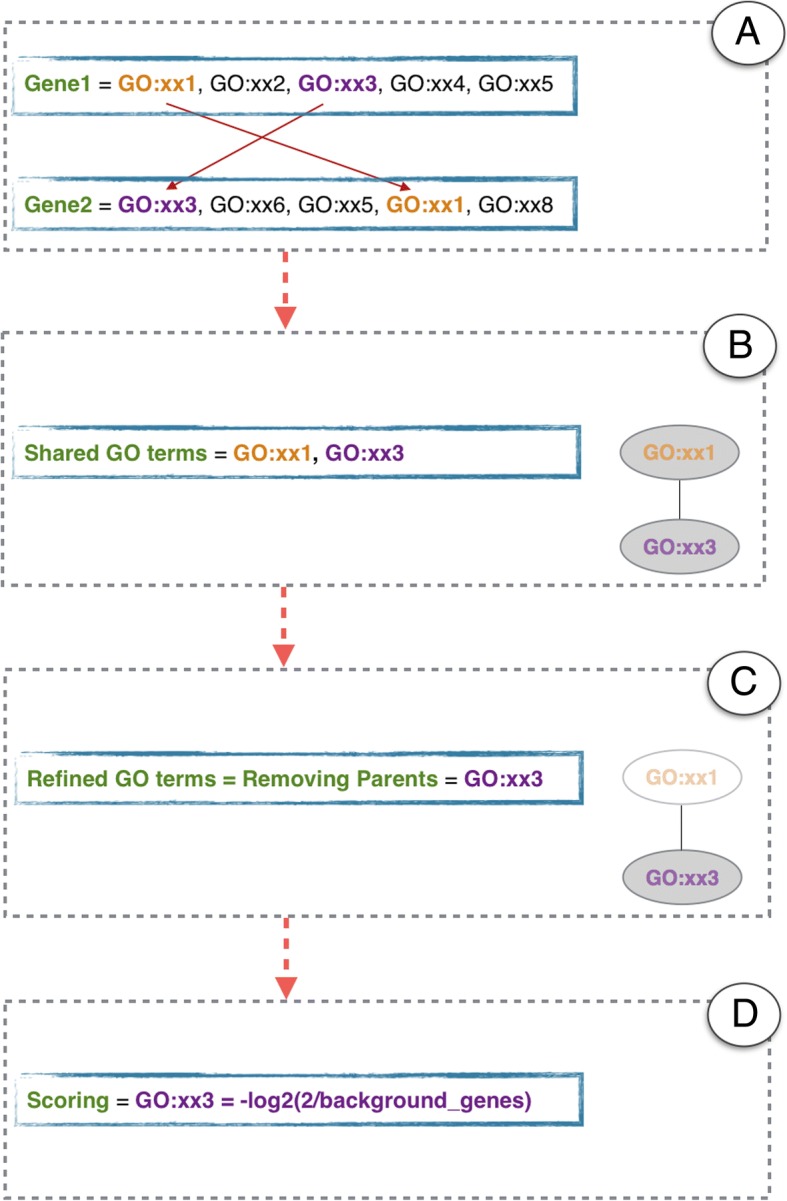
GOTrapper Algorithm. The algorithm is shown in Parts A-D. The GO terms of two genes are returned in Part A. The terms that are shared by the two genes remain in Part B. For the two returned shared terms, the one which is a parent term is removed in Part C. The refined shared term without a parent is scored in Part D

In this first part of the algorithm, the GO terms which are shared by the desired number of genes would be defined (Fig. 2). After that, any shared terms with one or more children are removed. This helps get the most specific GO terms. Namely, the last shared terms remain at the end. The tool makes use of an option called “Threshold” to allow the users to control and pick up different levels of the tree.

### Scoring of the resulting GO terms

After retaining the most specific shared terms, we applied a scoring system to provide more meaningful information to the user for ranking the GO terms. The terms are scored based on the negative log likelihood:

$$ Score(t)=-\log \left(p(t)\right) $$

where *p(t)* is calculated by:

$$ p(t)=\frac{2}{g(t)} $$

where the constant number of ‘2’ was assigned to it in the tool as the number of the minimum background genes in a shared GO term is two, and *g(t)* is the number of background genes, which is the total number of genes, annotated to the *t* term. The lower the number of background genes annotated to a term, the lower the *score(t)* would be. We assume that the lesser the *score(t)*, the more specific the term *t* is, as the number of annotated genes decreases in the terms going down the hierarchy.

### Threshold

The flexibility of GOTrapper increases by introducing the “Threshold” option. The minimum threshold is “2”, i.e.; the retained GO terms must be shared by at least two input genes. This option also provides the user with the ability to control the returned level (going up or down the tree) of the shared GO terms in the GO hierarchy by increasing or decreasing the threshold.

### Examples

We use different sets of genes [26, 27] to implement both functionalities (‘Within’ and ‘Between’) of GOTrapper.

### Grouping a list of genes using the “within” option

In using high-throughput microarray and next generation sequencing technologies, researchers compare the expression data for a large number of genes in two (or more) different states. Exemplary research was conducted on human prostate cancer using RNA-seq data [26], where malignant samples were compared with non-malignant. The study ended up with a large number of genes being expressed differently between the two conditions. The comparison held by the researchers resulted in a large number of GO terms with an exceedingly large number of background genes. The most common groups of GO terms achieved by the researchers were related to metabolic and cellular processes; which are known to be fundamental needs for the establishment of cancer. Other groups were related to regulation, development, nucleic acid binding, localization, biological adhesion, catalytic activity, structural molecule activity, immune response, and multicellular organism activity. We aim to compare a list of 815 differentially expressed genes (> = 2fold change) (Additional file 1), from the prostate cancer research mentioned above. We want to find possible associations among the genes and understand biological processes as well as molecular functions of the genes in highly specific terms based on the GO annotations. In this example, we used a threshold value of 10 (each GO term to be shared by at least 10 genes). Out of the total 1077 GO terms, which are shared among the respective genes, 319 most highly specific shared GO terms were trapped (Additional file 2). We classified the group of genes the same way discussed above. We could find a large number of genes related to regulation and developmental processes in the most specific GO terms. A large number of GO terms also allocate to metabolic and cellular processes. Nonetheless, genes associated with cell adhesion were rather so scarce. Other groups of GO terms met nicely with the classifications held by the researchers relating to prostate cancer. Indeed, after assigning our score scheme to the study, the terms got more specific and less redundant. This in turn aids in the easier and more efficient interpretation of biological data than when handling a large number of nonspecific redundant GO terms.

### Comparing two lists of genes using the “between” option

Here we want to compare two lists of genes. A list of six genes, known to be related to urea cycle disorders (CPS1, OTC, ASS1, ASL, ARG1, and NAGS) [27], is compared to a list of 114 Chromatin Remodeling genes (Additional file 3), which modify the chromatin architecture and make it accessible for transcription. Current research investigates how aberrant chromatin remodeling, among other epigenetic factors, is correlated with a wide spectrum of diseases [28]. Many diseases associated with chromatin remodeling are related to metabolism [29]. One such example of metabolic diseases is the urea cycle disorder. Current research has investigated the epigenetic modifications expected to be correlated with urea cycle disease [30]. We assume that a researcher has intention to investigate the correlation between the list of chromatin remodeler genes and the genes related to urea cycle disorder. Using this option to compare these two lists of genes, we find GO terms that are shared by at least one gene from each list by setting the threshold to two. This comparison resulted in 295 total shared GO terms and was refined to 72 highly specific shared terms (Table 1, Additional file 4). Interestingly, many shared GO terms between the two groups were associated with metabolism, including biosynthetic and catabolic processes. Many cellular processes are linked to the response to internal metabolites, including the ammonium ion, among others. Regulation involved metabolic processes associated with nitrogen compounds. Some abundant transport processes were also related to nitrogen compound transport. Additionally, response to amine stimulus was also involved in the set of GO terms. Many other processes were associated with development. Since the threshold was set to the minimum value, results are highly specific and the derived number of GO terms is much lesser. This could nicely help in supporting the hypothesis that urea cycle disease has a strong correlation with epigenetic modifications that can predispose as a result of, e.g., environmental factors.

**Table 1:** Top 10 terms shared by urea cycle disorder and chromatin remodeling genes

GO id	GO category	Background Genes	Genes	Score	GO term
GO:0071242	BP	27	CPS1, HDAC4	3.7549	cellular response to ammonium ion
GO:0045909	BP	29	CPS1, HDAC4	3.858	positive regulation of vasodilation
GO:0032964	BP	38	ARG1, ASL, ASS1, CPS1, NPM1, OTC, METTL3, NAGS	4.2479	collagen biosynthetic process
GO:0071398	BP	42	ARG1, ASS1, BNIP3, CPS1, HDAC2, HDAC5	4.3923	cellular response to fatty acid
GO:0014075	BP	47	BNIP3, CPS1, RB1, SIRT1	4.5546	response to amine stimulus
GO:0060416	BP	50	ARG1, ASS1, CPS1, HDAC2, KMT2A, OTC, HDAC4, HDAC5, CBX7	4.6439	response to growth hormone stimulus
GO:0055081	BP	53	ARG1, ASS1, CPS1, KAT2A, OTC, RB1, CHD8	4.7279	anion homeostasis
GO:0060135	BP	57	CPS1, OTC, CHMP3	4.8329	maternal process involved in female pregnancy
GO:1901655	BP	63	ASS1, CPS1, PHC1, HDAC2, SMARCD1	4.9773	cellular response to ketone
GO:0070301	BP	66	CPS1, OTC, CHMP3	5.0444	cellular response to hydrogen peroxide

## Results

We present GOTrapper; a methodology and user-friendly tool to devise new hypotheses and gene associations by going through the branches of the gene ontology tree by providing only the gene symbols or IDs.

The goal of GOTrapper is to assist researchers in finding gene associations and grouping the genes according to GO knowledge. A scoring system is provided to show the specificity of the terms. In addition, the tool allows the pre-selection of the evidence codes to be considered in the downstream analysis.

GOTrapper enables two types of input:

- ‘*Within*’: This option allows the input of a list of gene symbols or IDs to find the GO terms and association within this list (Fig. 1).
- ‘*Between*’: The purpose of this option is to find an association between two lists of genes in which the output GO terms have to be shared by at least two genes, each from a list (Fig. 1).

## Conclusions

GOTrapper is a user-friendly and multi-platform tool designed for experienced and non-bioinformaticians to cluster and group input genes of *Homo sapiens*. This allows the prediction of new hypotheses and helps find associations among the genes based on GO terms. Thus, the branches of the GO tree can be analyzed manually. The tool allows selection of the desired evidence code to be included in the process. A scoring system is also provided to determine the specificity of the returned GO terms.

### Availability of data and materials

**Project name**: GOTrapper.

**Project home page**: https://github.com/BioGeneTools/GOTrapper.

**Operating system(s)**: Platform-independent.

**Programming language**: Java.

**Other requirements**: Java (v1.7 or higher).

**License**: GNU GPL.

**Any restrictions to use by non-academics**: No.

## Additional files

Additional file 1: 815 DEGs from a prostate cancer study. (XLS 20 kb)

Additional file 2: 319 highly specific shared terms among 815 DEGs of the prostate cancer study. (TXT 693 bytes)

Additional file 3: 114 chromatin remodelers. (XLS 94 kb)

Additional file 4: 72 highly specific shared terms between 114chromatin remodelers and 6 urea cycle disorders. (TXT 5 kb)

## Abbreviation

GO: Gene ontology
